# Assessment of the efficacy and tolerability of ruxolitinib for the treatment of myelofibrosis patients in a real‐life setting: An Italian MYNERVA Project

**DOI:** 10.1002/cam4.5618

**Published:** 2023-01-27

**Authors:** Giacomo Coltro, Emanuela Sant'Antonio, Giuseppe A. Palumbo, Francesco Mannelli, Valerio De Stefano, Marco Ruggeri, Elena M. Elli, Roberta Zanotti, Oscar Borsani, Irene Bertozzi, Andrea Duminuco, Silvia Betti, Giuseppe Carli, Fabrizio Cavalca, Ilaria Tanasi, Elisa Rumi, Maria L. Randi, Bruno Garibaldi, Giuseppe G. Loscocco, Paola Guglielmelli, Alessandro M. Vannucchi

**Affiliations:** ^1^ Department of Experimental and Clinical Medicine University of Florence Florence Italy; ^2^ CRIMM, Center for Research and Innovation of Myeloproliferative Neoplasms Azienda Ospedaliero‐Universitaria Careggi Florence Italy; ^3^ GENOMEC Doctorate School, Medical Genetics, Department of Medical Biotechnologies University of Siena Siena Italy; ^4^ Department of Scienze Mediche Chirurgiche e Tecnologie Avanzate "G.F. Ingrassia" University of Catania Catania Italy; ^5^ Section of Hematology, Department of Radiological and Hematological Sciences Catholic University Rome Italy; ^6^ Dipartimento DIRE Fondazione Policlinico A. Gemelli IRCCS Rome Italy; ^7^ Hematology Department San Bortolo Hospital Vicenza Italy; ^8^ Hematology Division and Bone Marrow Transplantation Unit, San Gerardo Hospital ASST Monza Monza Italy; ^9^ Section of Hematology, Department of Medicine University Hospital of Verona Verona Italy; ^10^ Department of Molecular Medicine University of Pavia Pavia Italy; ^11^ Division of Hematology Fondazione IRCCS Policlinico San Matteo Pavia Italy; ^12^ First Medical Clinic, Department of Medicine‐DIMED University of Padua Padua Italy; ^13^ Postgraduate School of Hematology University of Catania Catania Italy

**Keywords:** efficacy, myelofibrosis, ruxolitinib, safety

## Abstract

**Background:**

Incorporating real‐world data in the drug development process allows the improvement of health outcomes by providing better representation of actual patterns of drug safety and efficacy.

**Aims and Methods:**

Here, we present the results of a retroprospective, observational real‐life study of 154 patients with myelofibrosis treated with ruxolitinib in a real‐life setting in seven Italian centers of the MYNERVA project.

**Results:**

Median drug exposure was 29 (range, 3–98) months. Discontinuation rate was 27% after a median time of 13 (range, 3–61). While hematological toxicities were in line with previous findings, infections occurred frequently, representing a not negligible cause of discontinuation and death. Anemia, symptoms, and spleen responses were obtained at any time in 23%, 91%, and 68% of patients, respectively; most patients achieved their responses by week 24. Larger splenomegaly and delayed treatment initiation correlated with lower spleen response at 24 weeks. Spleen response was associated with a superior overall survival, regardless of DIPSS. Of interest, both achievement and loss of spleen response had prognostic implications.

**Discussion and Conclusion:**

Overall, our findings provide insights on the efficacy and safety of ruxolitinib in a real‐world, multicenter cohort of Italian MF patients.

## Graphical abstract

This Brief Report presents the results of a real‐world study from the MYNERVA project that enrolled 154 patients with myelofibrosis treated with ruxolitinib. Hematological toxicities were consistent with previous studies, while infections occurred frequently, representing a not negligible cause of discontinuation and death. Anemia, symptoms and spleen responses were obtained by 23%, 91%, and 68% of patients, respectively. Both achievement and loss of spleen response had prognostic implications.

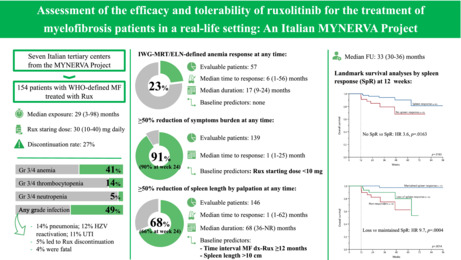

Ruxolitinib (Rux), the first JAK1/2 inhibitor approved for the treatment of myelofibrosis (MF), has been extensively tested in clinical trials. The phase III COMFORT studies, and their extended follow‐up, have demonstrated clinically meaningful and durable benefits in terms of reduction of splenomegaly and disease‐related symptoms resulting in overall improvement in health‐related quality of life in patients with intermediate‐2/high‐risk MF.^1^, ^2^, ^3^, ^4^, ^5^, ^6^ These results were confirmed in the phase IIIb, expanded‐access JUMP study that enrolled 2233 patients with intermediate‐1 to high‐risk MF, including those with thrombocytopenia and without splenomegaly.^7^

In the current study, we aimed at evaluating the efficacy and safety profile of Rux in a real‐life setting. We conducted a multicenter, retro‐prospective, observational study involving patients with World Health Organization‐defined MF who were treated with Rux in seven Italian tertiary centers referring to the MYNERVA project. Patients had to start Rux per commercial use between January 2015 and March 2018; alternatively, Rux could have been started within the JUMP trial^7^ and continued per commercial use following trial closure (September 2014). Response to Rux was evaluated according to the 2013 International Working Group‐Myeloproliferative Neoplasms Research and Treatment/European LeukemiaNet (IWG‐MRT/ELN) criteria^8^: spleen response (SpR) was assessed by palpation; symptom response (SyR) was defined dichotomically, as present or absent, according to physician's opinion. Survival probabilities, assessed from start of Rux to date of last follow‐up or death, were calculated using the Kaplan–Meier method and compared with the log‐rank test. Logistic regression and Cox regression model were used to calculate odds ratios (ORs) and hazard ratios (HRs), respectively.

## 1 CHARACTERISTICS OF THE STUDY COHORT

The study population included 154 patients, 66 (43%) primary and 88 (57%) secondary MF. Main baseline characteristics are summarized in Table 1. Median age at Rux start was 64 (35–81) years, and median time interval between MF diagnosis and Rux initiation was 16 (0–285) months; 84 (55%) patients were male. At baseline, hemoglobin <10 g/dl and platelets <100 × 10^9^/L were present in 51 (33%) and 19 (12%) patients, respectively; 139 (90%) patients referred constitutional symptoms, and median spleen length was 12 cm (1–33) from left costal margin, with 55% having a spleen length >10 cm. DIPSS risk categories were intermediate‐1 in 66 (43%) patients, intermediate‐2 in 78 (51%), and high in 10 (6%).

**TABLE 1:** Main characteristics of the 154 patients with myelofibrosis included in the study

Parameter	Value
Male sex; no (%)	84 (55)
Median age at MF diagnosis; years (range)	64.2 (35.8–81.0)
Median age at Rux start; years (range)	66.6 (44.3–81.7)
Age >65 years at Rux start; no (%)	96 (62)
MF type	
Primary MF; no (%)	66 (43)
Post‐polycythemia vera MF; no (%)	50 (33)
Post‐essential thrombocytemia MF; no (%)	38 (25)
Mutational status	
*JAK2* ^V617F^ mutated; no (%)	122 (79)
Median *JAK2* ^V617F^ allele burden; % (range)	71.0 (0.4–100)
*CALR* mutated; no (%)	21 (15)
*MPL* mutated; no (%)	4 (3)
Triple negative; no (%)	6 (4)
Unknown; no (%)	1 (1)
DIPSS risk category	
Intermediate‐1; no (%)	66 (43)
Intermediate‐2; no (%)	78 (51)
High; no (%)	10 (7)
Median WBC; ×10^9^/L (range)	12.6 (1.6–169.0)
WBC >25 × 10^9^/L; no (%)	26 (17)
Median hemoglobin; g/dl (range)	10.9 (5.7–15.6)
Hemoglobin <10 g/dl; no (%)	51 (33)
Median platelet; ×10^9^/L (range)	261 (49–1307)
Platelet <100 × 10^9^/L; no (%)	19 (12)
Constitutional symptoms; no (%)	139 (90)
Palpable spleen; no (%)	198 (97)
Spleen >10 cm from LCM; no (%)	84 (55)
Transfusion dependence; no (%)	25 (16)
Rux starting dose	
20 mg BID; no (%)	71 (46)
15 mg BID; no (%)	36 (23)
10 mg BID; no (%)	19 (12)
15 mg QD; no (%)	2 (1)
5 mg BID; no (%)	26 (17)
Median time between MF diagnosis to Rux start; months (range)	15.9 (0.0–285.4)
Time between MF diagnosis and Rux start ≥12 months; no (%)	62 (40)

Abbreviations: DIPSS, dynamic international scoring system; LCM, left costal margin; MF, myelofibrosis; Rux, ruxolitinib; WBC, white blood cell.

## 2 RUXOLITINIB DOSING AND DISCONTINUATION

Median exposure to Rux was 29 (3–98) months. Median starting dose was 30 (10–40) mg daily. Most patients (76%) needed a dose modification during the treatment. A total of 101 (66%) patients required ≥1 dose reduction, most frequently due to thrombocytopenia (49%) and anemia (23%), while 46 (30%) were able to increase Rux at least once; in particular, 40% and 50% of patients who started Rux at a total daily dose of ≤20 and ≤10 mg, respectively, needed a dose increment, mostly due to suboptimal spleen and/or symptom response.

At last follow‐up, 112 (73%) were still on Rux, and 42 (27%) discontinued treatment after a median of 13 (3–61) months. Most frequent reasons for discontinuation included hematopoietic stem cell transplantation (24%), infection, loss of response, progressive disease, and death (12% each). The rate of discontinuation was significantly higher in patients with DIPSS high (50%) compared to intermediate‐2 (32%) and intermediate‐1 (18%) risk. Of note, the most frequent reason for discontinuation among patients with DIPSS high was leukemic transformation (LT; 40%). Since this study was conducted long before the availability in Italy of alternative JAK‐inhibitors, we speculate that the discontinuation rate would be nowadays higher, since most patients were maintained with Rux despite partial response owing to the lack of alternatives.

## 3 SAFETY PROFILE

New‐onset or worsening grade 3/4 anemia, thrombocytopenia, and neutropenia were reported at some point during treatment in 41%, 14%, and 5% of patients, respectively. No patients discontinued Rux due to anemia, while three did so due to thrombocytopenia. Forty‐eight (32%) patients received red blood cell transfusion after a median of 3 (0–47) months, while five (3%) needed at least one platelet transfusion. Infections occurred in 49% of patients after a median of 8 (0–58) months; the most frequent were pneumonia (14%), herpes‐zoster reactivation (12%), and urinary tract infection (11%). In seven (5%) patients the infection led to treatment discontinuation, in six (4%) was fatal (two pneumonia, three septic shocks, one pulmonary aspergillosis).

## 4 EFFICACY PROFILE

An IWG‐MRT/ELN‐defined anemia response (AR) was achieved by 13 (23%) of 57 evaluable patients after a median of 6 (1–56) months; no pre‐treatment factors were found to predict AR achievement. Median duration of AR was 17 (9–24) months, and 3 (23%) patients lost their response. Full interpretation of these findings is prevented by lack of annotation for concomitant medications addressing MF‐associated anemia.

A SyR was achieved by 126 (91%) of 139 evaluable patients after a median of 1 (1–25) month, with SyR at 24 weeks (SyR_24_) being reported in 125/126 patients. In univariate analysis, only Rux starting dose <10 mg was found to correlate with lower probability of obtaining SyR at any time (*p* = 0.0443).

Among 146 (95%) evaluable patients, 100 (68%) experienced an IWG‐MRT/ELN‐defined SpR at any time, with median time to SpR of 1 (1–62) month. Most patients (97/100) achieved SpR at week 24 (SpR_24_). A SpR_24_ was reported in 75% of patients with intermediate‐1 MF compared to 60% of intermediate‐2/high‐risk disease (*p* = 0.07). In univariate analysis, baseline factors negatively correlating with SpR_24_ were time interval between MF diagnosis and Rux initiation ≥12 months (*p* = 0.011), baseline spleen length >10 cm (*p* = 0.0049), DIPSS intermediate‐1 versus intermediate‐2/high (*p* = 0.05), and Rux starting dose <40 mg daily (*p* = 0.0387). Upon multivariate analysis, time interval ≥12 months (OR 2.7, 95% confidence interval [CI] 1.3–5.7; *p* = 0.0112) and baseline spleen length >10 cm (OR 2.8, 95% CI 1.3–6.1; *p* = 0.0078) were confirmed to be independent predictors of inferior SpR_24_. At last follow‐up, 34 (34%) patients lost their SpR, with median duration of SpR of 68 (36‐not reached) months. Univariate Cox analysis identified primary (vs. secondary) MF (*p* = 0.0365), platelets <100 × 10^9^/L (*p* = 0.0193) and Rux starting dose <10 mg (*p* = 0.0007) as pre‐treatment variables predicting higher risk of SpR loss.

A total of 6 (4%) patients failed to achieve any IWG‐MRT/ELN‐defined responses and were defined primary resistant. Among baseline factors, platelets <100 × 10^9^/L (*p* = 0.0140) and splenomegaly >10 cm were associated with a higher probability of primary resistance.

## 5 OUTCOMES

**FIGURE 1:**
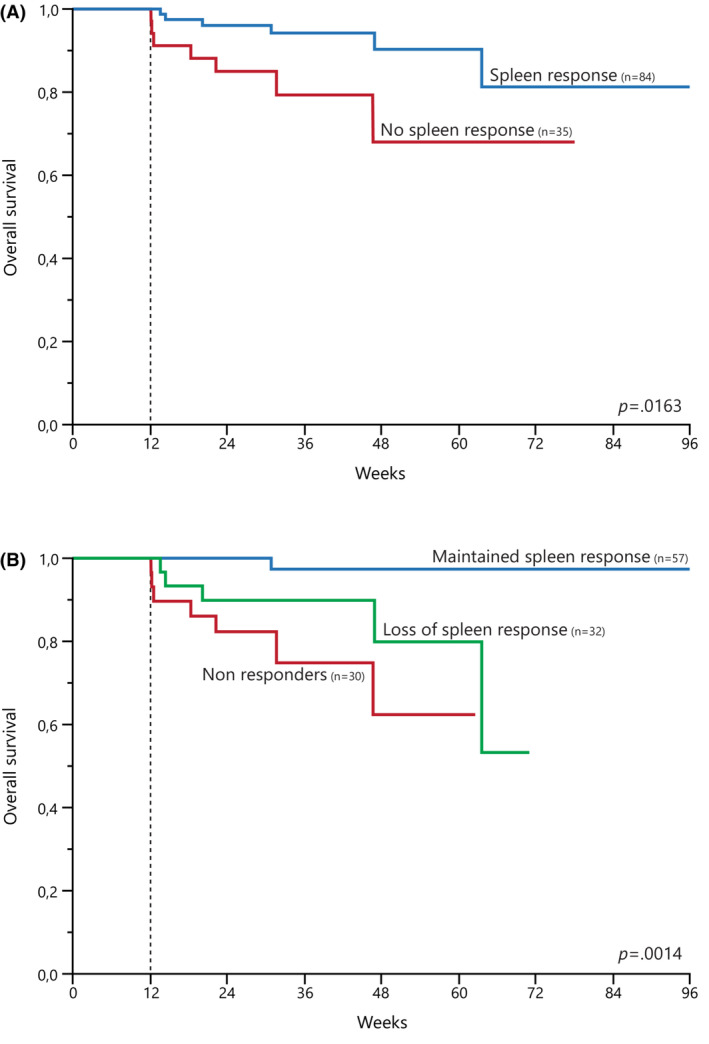
Landmark survival analyses by spleen response at 12 weeks. (A) Kaplan–Meier estimates of overall survival by the achievement of spleen response. (B) Kaplan–Meier estimates of overall survival in patients that maintained spleen response, achieved but lost spleen response, and never obtained spleen response.

After a median follow‐up of 33 (30–36) months, 18 (12%) deaths and 7 (5%) LTs were recorded. Due to the short follow‐up, median overall survival (OS) was not reached. By landmark survival analysis, SpR was associated with superior OS (HR 3.6, 95% CI 1.2–10.8, *p* = 0.0163; Figure 1A). This survival advantage was retained when OS estimations were adjusted for the DIPSS (intermediate‐1 vs. intermediate/high risk). We then assessed the impact of SpR loss, and found that OS of patients who lost their SpR was significantly worse compared to patients who maintained the response (HR 9.7, 95% CI 1.2–82.6, *p* = 0.0004), and not significantly different in comparison to non‐responders (*p* = 0.2; Figure 1B).

Data from clinical trials carry intrinsic methodological caveats that may limit their reproducibility in the everyday clinical practice, particularly as regards safety and durability of outcomes. In this study, we report a multi‐institutional cohort of MF patients treated with Rux in a real‐life setting with the aims to provide independent data on the efficacy and tolerability. The prevalence of grade 3/4 anemia and thrombocytopenia was consistent with previous studies,^1^, ^2^, ^3^, ^4^, ^5^, ^6^, ^7^ and rarely lead to discontinuation. Infections occurred in almost half of the patients, especially pneumonia, herpes‐zoster reactivation, and urinary tract infection, led to Rux discontinuation in seven patients, and were fatal in six. Compared to previous studies,^9^ our real‐life observations revealed a higher rate of infections, although the lack of severity grading prevents full interpretation of the data. Nevertheless, these findings underscore the need of a careful assessment of individual infectious risk when planning Rux treatment in a MF patient. Majority of patients experienced some reduction in spleen length during Rux treatment, with 66% achieving a palpable SpR_24_. Such a proportion is significantly higher compared to COMFORT trials (32%–54%),^1^, ^2^, ^3^, ^4^, ^5^, ^6^ but is consistent with subsequent studies^7^, ^10^ and real‐life experiences^11^, ^12^, ^13^ that used palpation to evaluate splenomegaly and included patients with intermediate‐1 risk MF. Of interest, baseline spleen length >10 cm and time interval between MF diagnosis and Rux start were independently associated with a lower probability of SpR_24_, in line with previous findings.^14^ We showed that patients achieving SpR had better survival estimates compared to non‐responders, independent of DIPSS. Of note, our findings confirm the adverse prognosis of patients who lost their response.^15^, ^16^ Finally, consistent with post‐marketing studies,^7^, ^10^ our data support the satisfactory performance of Rux in patients with DIPSS intermediate‐1 risk MF.

In conclusion, despite study limitations (i.e., retro‐prospective design, small cohort, short follow‐up, reporting biases, inconsistency of subjective assessment of SyR), this analysis provides insights on the efficacy and safety of Rux in a real‐world, multicenter cohort of Italian MF patients. Most relevant findings are (i) high incidence of infectious complications, that resulted fatal in a non‐negligible proportion of cases; (ii) confirmation of a remarkable symptomatic activity of Rux; and (iii) the prognostic implications of both the achievement and the loss of response to Rux.

## AUTHOR CONTRIBUTIONS

**Giacomo Coltro:** Data curation (equal); formal analysis (lead); investigation (equal); project administration (equal); writing – original draft (equal); writing – review and editing (equal). **Emanuela Sant'Antonio:** Conceptualization (equal); writing – review and editing (equal). **Giuseppe A. Palumbo:** Data curation (equal); writing – review and editing (equal). **Francesco Mannelli:** Data curation (equal); writing – review and editing (equal). **Valerio De Stefano:** Data curation (equal); writing – review and editing (equal). **Marco Ruggeri:** Data curation (equal); writing – review and editing (equal). **Elena M. Elli:** Data curation (equal); writing – review and editing (equal). **Roberta Zanotti:** Data curation (equal); writing – review and editing (equal). **Oscar Borsani:** Data curation (equal); writing – review and editing (equal). **Irene Bertozzi:** Data curation (equal); writing – review and editing (equal). **Andrea Duminuco:** Data curation (equal); writing – review and editing (equal). **Silvia Betti:** Data curation (equal); writing – review and editing (equal). **Giuseppe Carli:** Data curation (equal); writing – review and editing (equal). **Fabrizio Cavalca:** Data curation (equal); writing – review and editing (equal). **Ilaria Tanasi:** Data curation (equal); writing – review and editing (equal). **Elisa Rumi:** Data curation (equal); writing – review and editing (equal). **Maria L. Randi:** Data curation (equal); writing – review and editing (equal). **Bruno Garibaldi:** Data curation (equal); writing – review and editing (equal). **Giuseppe G. Loscocco:** Data curation (equal); writing – review and editing (equal). **Paola Guglielmelli:** Conceptualization (equal); funding acquisition (equal); writing – original draft (equal); writing – review and editing (equal). **Alessandro M. Vannucchi:** Conceptualization (equal); funding acquisition (equal); methodology (equal); project administration (equal); supervision (equal); writing – original draft (equal); writing – review and editing (equal).

## FUNDING INFORMATION

This project has been supported by grants from Associazione Italiana per la Ricerca sul Cancro‐AIRC, project 5 per Mille MYNERVA (MYeloid NEoplasms Research Venture AIRC), code 1267; by the Italian Ministry of Health, project Ricerca Finalizzata, code NET‐2018‐1236593; and by a donation form Famiglia Maria Cuomo.

## CONFLICT OF INTEREST

A.M.V. has received speaker fees from Novartis, AOP Health, Incyte, AbbVie, GlaxoSmithKline (GSK), and Bristol Myers Squibb (BMS); and has participated to the advisory boards of Novartis, Incyte, AOP Orphan Pharmaceuticals, AbbVie, GSK, BMS, and Roche. E.R. has been a consultant to Novartis. G.A.P. has received speaker fees from AbbVie, Bristol Myers Squibb (BMS), Incyte, Novartis, has participated in advisory boards of AbbVie, AOP, Orphan Pharmaceuticals, AstraZeneca, BMS, Novartis, and received support for attending meetings from AbbVie, BMS, Jannsen, Novartis. P.G. has received speaker fees from AbbVie and Novartis, and support for attending meetings from Sanofi. V.D.S. has received speaker fees from and has participated to the advisory boards of AbbVie, AOP Health, Bristol Myers Squibb, and Novartis. The other authors have no competing interest to declare.

## ETHICAL APPROVAL

The study was conducted in accordance with the Declaration of Helsinki and European and Italian regulations, and was approved by the ethical committees of each institution prior to the start of the study. Central approval of the MYNERVA project was from Florence IRB, #14560.

## INFORMED CONSENT

Written informed consent was obtained from the participating subjects prior to study entry.

## Data Availability

The datasets used and/or analyzed during the current study are available from the corresponding author on reasonable request.
